# Editorial for the Special Issue: The Role of the Virome in Health and Disease

**DOI:** 10.3390/microorganisms10010020

**Published:** 2021-12-23

**Authors:** Felix Broecker

**Affiliations:** Idorsia Pharmaceuticals Ltd., Hegenheimermattweg 91, CH-4123 Allschwil, Switzerland; felixbroecker@gmx.net

Viruses are the most abundant biological entities on Earth, with an estimated total of 10^31^ virions [1]. About 140,000 viral species reside in our intestine [2], at least 200,000 are found in the oceans [3], and many more in other habitats. Of these, only about 200 virus species are known to cause disease in humans, including the well-known ebolaviruses, human immunodeficiency virus (HIV), and influenza viruses [4]. In 2019, a new virus was added to this list: severe acute respiratory syndrome coronavirus type 2 (SARS-CoV-2). Originally most likely a bat virus, it entered the human population via a presently unknown route. Ebolaviruses are also likely to originate from bats [5], HIV originated from chimpanzees [6], and the 2009 H1N1 pandemic influenza virus was traced back to pigs [7]. Yet, these viruses that infect humans and cause disease are only a tiny fraction, less than 0.1%, of the total number of viruses we know to exist on Earth; the vast majority of viruses do not cause us any harm. Some are even known for their beneficial functions, such as training of our immune system [8,9]. Bacterial viruses, bacteriophages, can be used therapeutically, and could help us to alleviate the ‘antibiotic crisis’ [10]. Thus, the impact of viruses on our health covers the entire spectrum, from deadly pathogens to life-saving agents.

There is another type of viruses: those that during evolution have been incorporated into the genomes of us and other species, known as endogenous viruses. Most of them are remnants of retroviral proviruses and close relatives of viruses—transposable elements. Their roles are also ambivalent. Due to their overall activation in cancer, they are sometimes labeled as the ‘enemy within’ [11]. Yet, endogenous viruses also contribute to the evolution of our immune systems and provide us and other mammals with the ability to give live birth, among other abilities [12]. The emerging view is that, as is the case with bacteria, viruses are an integral part of the (human) ecosystem, and the majority can be regarded as commensals with no substantial positive or negative effect on health [13]. Yet, we are only beginning to understand the composition, not to mention the biological consequences, of the virome. A large proportion of viromes are still undefined ‘viral dark matter’—virus-like sequences without similarity to any known taxon [14]. It was my intention with this Special Issue to shed some light on the various aspects of the virome with a focus on the impacts on human health and disease.

Łusiak-Szelachowska and colleagues provide a comprehensive overview of the multifaceted roles of bacteriophages [15]. The authors point out that the ‘phageome’ is pathologically altered in various diseases such as inflammatory bowel disease, type 1 diabetes and malnutrition. These authors also highlight the potential of phage intake to ameliorate some of the symptoms associated with such diseases. Staying in the realm of phages, Hedžet et al. report the discovery of *Bacteroides*-infecting phages from the human intestine that contain a diversity-generating retroelement (DGR) [16]. First identified in *Bordetella* phage BPP-1, DGRs appear to be evolutionarily co-opted by some phages to generate variation in the proteins required for the infection of the host bacteria, thereby altering or extending the phage’s tropism [17]. This study shows that the identified *Bacteroides*-phages also utilize their DGR in a similar manner, pointing towards a more widespread mechanism in phages.

Yuan et al. shed some light on the aforementioned ‘enemy within’, particularly human endogenous retroviruses (HERVs), in glioblastoma multiforme (GBM) [18]. GBM is a highly aggressive and common form of brain tumor. The authors studied the expression levels of various HERVs in GBM in great detail and found various HERV types to be aberrantly expressed, revealing, for example, novel potential biomarkers or therapeutic targets. Indeed, not only endogenous, but various exogenous viruses have confirmed or suspected roles in various types of cancer. Together with Karin Moelling, we wrote a review—to our knowledge, the most comprehensive one—on the various roles of viruses in cancer formation [19]. We discuss well-known oncogenic viruses, such as human papillomaviruses, but also the more recently unveiled roles of phages in cancer formation, as well as the responsiveness to cancer immunotherapy. We expect lots of new such associations to be discovered soon and perhaps phages will be able to help us in the fight against cancer.

Where should we look for new viruses? Any habitat on Earth harbors plenty of them. For example, the article by Rumbou et al. presents, to my understanding, the most comprehensive overview of the forest virome [20]. Such data will, among other findings, allow for better pathogen control and management, and thereby help preserve precious ecosystems. I am certain that with the rapidly advancing sequencing and bioinformatic pipelines, we will make substantial progress in deciphering the ‘viral dark matter’, with a significant positive impact on global health.
